# Assessing the association between cholinergic integrity and cognitive performance in adults with Down Syndrome using cholinergic anatomy and [^18^F]‐FEOBV PET

**DOI:** 10.1002/alz.091867

**Published:** 2025-01-09

**Authors:** Jason K Russell, Alexander C Conley, Brian Boyd, Rachel Schlossberg, Allison Stranick, Adam J Rosenberg, Lealani Mae Y. Acosta, Michael Rafii, Sepideh Shokouhi, Paul A Newhouse

**Affiliations:** ^1^ Center for Cognitive Medicine, Vanderbilt University Medical Center, Nashville, TN USA; ^2^ Vanderbilt University Medical Center, Nashville, TN USA; ^3^ Alzheimer's Therapeutic Research Institute, University of Southern California, San Diego, CA USA; ^4^ GRECC, VA, Tennessee Valley Healthcare System, Nashville, TN USA

## Abstract

**Background:**

Triplication of the amyloid precursor protein in individuals with Down Syndrome (DS) produces an increased risk for the development of Alzheimer's disease (AD). Declining cholinergic integrity plays a role in the cognitive deficits observed in late‐onset AD. In the present study, we assess the relationship between basal forebrain volume or [^18^F]‐FEOBV uptake and cognitive performance in adults with DS.

**Method:**

188 individuals from the ABC‐DS cohort (U01AG051406; U01AG051412; U19AG068054) were assessed cross‐sectionally. Basal forebrain volumes were calculated from T1 MRIs using the ScLimbic FreeSurfer pipeline. Ten individuals were recruited as part of a TRC‐DS sub‐study (25‐55 years old) or de novo (18‐24 years old), where they underwent MRI imaging and [^18^F]‐FEOBV PET imaging. Regional [^18^F]‐FEOBV SUVRs were calculated using PetSurfer. Participants from both studies underwent numerous cognitive assessments. Linear regressions were performed between cognitive performance and regional [^18^F]‐FEOBV SUVR or basal forebrain volume correcting for intellectual disability.

**Result:**

In the ABC‐DS cohort, the Down Syndrome Mental State Exam (DSMSE) total score was positively associated with basal forebrain volume (p<0.05), and total free recall on the cued recall task was positively associated with left basal forebrain volume (p=0.0027). In the TRC‐DS sub‐study, [^18^F]‐FEOBV uptake in the right amygdala, right rostral anterior cingulate cortex, and left transverse temporal cortex were positively associated with DSMSE total score (p<0.05). In the cued recall task, [^18^F]‐FEOBV uptake in the left cuneus and right entorhinal cortexes and the isthmus of the right cingulate cortex was negatively associated with total free recall (p<0.05).

**Conclusion:**

In the ABC‐DS cohort, increased cholinergic integrity predicted improved performance on the DSMSE and cued recall task. Regional [^18^F]‐FEOBV uptake can indicate which areas of cholinergic innervation are important for these different cognitive tasks. Increased regional [^18^F]‐FEOBV uptake predicted improved performance on the DSMSE, with the object memory and apraxia sub‐scores displaying the largest effect sizes. Interestingly, increased [^18^F]‐FEOBV uptake predicted decreased free recall in the cued recall task. This unexpected result may indicate upregulation of the vesicular acetylcholine transporter in this non‐demented DS cohort or may be spurious due to the small cohort. Increased sample size will further elucidate this effect.

